# Pan-American Medical Congress

**Published:** 1892-04

**Authors:** Charles A. L. Reed, J. W. Carhart

**Affiliations:** Chairman; Secretary


### Society Notes.

### THE PAN-AMERICAN MEDICAL CONGRESS.

The Committee on Permanent Organization met at St. Louis, October 14, 15 and 16, 1891, and adopted a series of general regulations for the permanent organization of the Pan-American Medical Congress, and a series of special regulations for the government of the first meeting, and recommended that the incorporators adopt both series of regulations as the organic law of the Congress.

Pursuant to such regulations, the following general officers were elected, viz.:

Wm. Pepper, M. D., LL. D., Philadelphia, Pa., President.

Abraham M. Owen, A. M., M. D., Evansville, Ind., Treasurer.

Charles A. L. Reed, M. D., Cincinnati, O., Secretary General.

[International Executive Committee omitted.—Ed.]

The Auxiliary Committee nominated by the various members of the Committee on Permanent Organization, each for his own State, and already commissioned by the chairman, was confirmed.

The election of officers of sections was begun, but time would not permit of the completion of the list, which was referred to a special committee with power to act. It has been deemed inexpedient to publish the list until it is completed, which can hardly be accomplished before the meeting of the Committee on Permanent Organization, in Detroit, in June; but the organization of particular sections will be announced through the medical press as rapidly as officers are elected by the special committee.

In accordance with the wish of the Committee on Permanent Organization, as expressed in special regulation No. 4, Drs. I. N. Love, A. B. Richardson, L. S. McMurtry, R. B. Hall, T. V. Fitzpatrick and Charles A. L. Reed met in Cincinnati and signed the legal form of application for articles of incorporation of the Pan-American Medical Congress, which articles of incorporation were duly issued by the Secretary of the State of Ohio, under date of March 15, A. D. 1892.

At a meeting of the incorporators held March 16, 1892, the following regulations, general and special, recommended by the Committee on Permament Organization, were formally adopted as the organic law of the Pan-American Medical Congress, in accordance with the laws of Ohio, and all elections had by the Committee on Permanent Organization, in accordance with such regulations were confirmed, and made a part of the laws of the Congress:

[General regulations omitted; a copy can be had, free of charge, by writing to Secretary Chas. A. L. Reed, Cincinnati.—Ed.]

SPECIAL REGULATIONS OF THE FIRST CONGRESS.—TIME AND PLACE OF MEETING.

1. The first Pan-American Medical Congress shall be held in the city of Washington, D. C., September 5, 6, 7, 8, A. D. 1893.

REGISTRATION.

2. The registration fee shall be $10 for members residing in the United States, but no fee shall be charged to foreign members. Each registered member shall receive a card of membership, and be furnished a set of the transactions.

[The balance of special regulations omitted for want of space. Apply to Secretary for copy.—Ed.]

Pursuant to the laws of Ohio and the regulations adopted as above, and in accordance with nominations by the Committee on Permanent Organization, the incorporators elected fifteen trustees, as follows:

Dr. W. T. Briggs, Tennessee; Dr. Geo. F. Shrady, New York; Dr. P. O. Hooper, Arkansas; Dr. S. S. Adams, D. C.; Dr. H. O. Marcy, Massachusetts; Dr. J. F. Kennedy, Iowa; Dr. H. D. Holton, Vermont; Dr. L. S. McMurtry, Kentucky; Dr. N. S. Davis, Illinois; Dr. Levi Cooper Lane, California; Dr. I. N. Love, Missouri; Dr. Hunter McGuire, Virginia; Dr. J. C. Culbertson, Illinois; Dr. A. Walter Suiter, New York; Dr. C. H. Mastin, Alabama.

Drs. L. S. McMurtry (Ky.), I. N. Love (Mo.) and W. W. Potter (N. Y.), were designated to act as members of the Executive Committee.

The organization of the Congress is complete in British North America, the British West Indies, the Spanish West Indies, Guatemala, Nicaragua, United States of Columbia, Brazil, Uruguay, Venezuela and the Argentine. It is confidently expected that the nominations from the remaining counties will be in by June.

It is expected to announce the completed organization of the

Congress, its sections and auxiliary committees, domestic and foreign, by July 1, 1892.

On behalf of the Committee on Permanent Organization,

CHARLES A. L. REED, Chairman.

J. W. CARHART, Secretary.

